# Metabolome and transcriptome integration explored the mechanism of browning in *Glycyrrhiza uralensis* Fisch cells

**DOI:** 10.3389/fpls.2024.1305871

**Published:** 2024-07-09

**Authors:** Xinyang Zhao, Xueshuang Li, Aodun Bao, Xiaoli Zhang, Yongbin Xu, Yali Li

**Affiliations:** School of Life Science and Technology, Inner Mongolia University of Science and Technology, Baotou, China

**Keywords:** browning cells, UPLC-MS, RNA-Seq, flavonoids synthesis, *Glycyrrhiza uralensis* Fisch

## Abstract

**Introduction:**

*Glycyrrhiza uralensis* Fisch, a traditional Chinese medicinal herb known for its diverse pharmacological effects including heat-clearing, detoxification, phlegm dissolving, and cough relief, has experienced an exponential increase in demand due to its expanding clinical use and development prospects. Currently, large-scale cell culture stands out as one of the most promising biotechnological approaches for producing bioactive compounds from medicinal plants. However, the problem of cell browning represents a significant bottleneck in industrial applications of cell culture.

**Methods:**

This study focuses on the *Glycyrrhiza uralensis* Fisch cells from the Ordos plateau, aiming to elucidate the enzymatic browning process during plant cell culture. Key substrates and genes involved in enzymatic browning were identified by metabolome and transcriptome analysis of normal and browning cells.

**Results:**

Metabolome analysis reveals significant changes in the levels of chalcone, isoflavone, imidazole-pyrimidine, purine nucleosides, organic oxides, carboxylic acids and their derivatives, benzene and its derivatives, flavonoids, 2-arylated benzofuran flavonoids, diazanaphthalenes and fatty acyls within browning cells. In particular, chalcones, isoflavones, and flavones compounds account for a higher proportion of these changes. Furthermore, these compounds collectively show enrichment in four metabolic pathways: Isoflavone biosynthesis pathway; Cutin suberine and wax biosynthesis pathway; Aminoacyl-tRNA biosynthesis pathway; Isoquinoline alkaloid biosynthesis pathway; Transcriptome analysis revealed that the MYB transcription factor is a key regulator of flavonoid synthesis during the browning process in cells. In addition, 223 differentially expressed genes were identified, including phenylpropane, shikimic acid, glycolysis, and pentose phosphate pathways. Among these genes, 23 are directly involved in flavonoid biosynthesis; qPCR validation showed that eight genes (*GlPK, GlPAL, Gl24CL, Gl1PDT, Gl3CHI, GlC4H, Gl2F3’H*, and *Gl2CCR*) were up-regulated in browning cells compared to normal cells. These findings corroborate the sequencing results and underscore the critical role of these genes in cellular browning.

**Discussion:**

Consequently, modulation of their expression offers promising strategies for effective control of cellular browning issues.

## 1 Introduction

*Glycyrrhiza uralensis* Fisch(*G. uralensis*), a perennial herbaceous plant belonging to the legume family (Parvaiz et al., 2014), contains over 400 compounds, including triterpenoids, flavonoids, isoflavones, and phenolic compounds (Wu et al., 2022). It shows potential effects such as heat-clearing and detoxification, cough relief, and phlegm dissolving (Ren et al., 2023), and harmonizing various drugs. Moreover, it possesses antibacterial, anti-inflammatory, antioxidant, antiviral, hepatoprotective, and anti-tumor activities (Adhikari et al., 2021; Kim et al., 2021). Glycyrrhizin is extensively used as a natural sweetener in the food and tobacco industries( (Mochida et al., 2017). Currently, there is an increasing demand for the application of plant secondary metabolites, but wild *G. uralensis* resources are declining in yield with limited availability. Artificial cultivation of *G. uralensis* faces challenges such as varietal degradation and high costs. Large-scale cell culture opens up new avenues to address *G. uralensis* resource issues (Roberts and Shuler, 1997). Nevertheless, this technology still faces several challenges during the industrialization process, including biological and engineering problems. However, the issue of cell browning throughout culture is very important.

Browning phenomena can be categorized into non-enzymatic browning and enzymatic browning (Techakanon et al., 2017). Non-enzymatic browning reactions mainly occur during food preparation and storage processes (Croguennec, 2016), encompassing four pathways: polyphenol oxidation condensation reaction, Maillard reaction, oxidative decomposition of ascorbic acid, and caramelization (Das et al., 2022), which is critical for food and affects food stability and nutritional value (Manzocco et al., 2000; Ye et al., 2023). Some external factors (temperature, amino compounds (Lu et al., 2014), pH, water activity, and metal ions (Haleva-Toledo et al., 1997; Carabasa-Giribet and Ibarz-Ribas, 2000)that can cause changes in foods color are also non-enzymatic browning (Ahad et al., 2020). Enzymatic browning is a process in which phenols are catalyzed by polyphenol oxidase (PPO) to form quinones and their polymers (Martinez and Whitaker, 1995). During the normal developmental process of plant cells, PPO is primarily located in the chloroplasts, while phenolics are found in the vacuoles (Vaughn and Duke, 1984; Coetzer et al., 2001). They are situated in different regions of the cell and do not interact with each other, so browning does not occur during normal plant development. However, when plant cells are damaged, the enzyme binds with phenolics and then undergoes an oxygen-induced enzymatic browning reaction (Vaughn et al., 1988). Phenolics, as substrates of PPO, have a complex structure and are precursors for the synthesis of flavonoids, lignins, tannins, isoflavonoids, and derivatives of the phenylpropane pathway in plants (Cheynier, 2012), with a major flux towards both lignin metabolism and flavonoid metabolism. Recent research has identified enzymatic browning as the primary cause of browning in plant cell cultures.

In recent years, the advent of high-throughput sequencing technology has propelled RNA-seq, a next-generation transcriptome sequencing technique based on the Illumina platform, to become the preferred method for quantifying gene expression levels (Mortazavi et al., 2008). Metabolome involves a comprehensive assessment of endogenous metabolites (Zhang et al., 2012), and aims to provide an exhaustive and precise analysis of all low-abundance metabolites. It also serves as a complementary approach to other omics technologies (Heyman and Dubery, 2016). By transcriptome and metabolome integrating analyses to qualitatively and quantitatively study metabolic changes and metabolic pathways in organisms, identify genes associated with browning occurrence, and comprehensively unravel the cascade from gene expression to metabolite formation (Hao et al., 2022).

Normal and browning *G. uralensis* cells were used as research materials after solid-liquid transformation and metabolome and transcriptome analysis were conducted using UPLC-MS and RNA-seq technology to identify differential metabolites, metabolic pathways and differentially expressed genes that cause enzymatic browning of cells. Ultimately, regulation of gene expression can provide a foundation for controlling cell browning that occurs after solid-liquid transformation.

## 2 Materials and methods

### 2.1 Donor plant materials

*G. uralensis* seeds were generously provided by Inner Mongolia Yili Licorice Co., Ltd. Firstly, seeds underwent seed coat crushing and disinfection treatment, following these specific steps: soaking in 98% H_2_SO_4_ for 35 minutes, distilled water rinsing 5 times; Soaking in 70% ethanol for 3 minutes, distilled water rinsing 5 times; Sterilizing 0.1% HgCl_2_ for 1 min, distilled water rinsing 5 times. Subsequently, the treated seeds were inoculated onto solid Murashige and Skoog (MS) medium (Murashige and Skoog, 1962) supplemented with 30g L^-1^ sucrose, 1g L^-1^ inositol, 0.8% (w/v) agar and placed in a light culture box at a temperature of 26°C with a light cycle of 16 hours per day for 2 weeks.

### 2.2 Plant callus culture

Each cotyledon and hypocotyl of the donor seedlings were used as explants for callus induction and inoculated into solid MS medium (Murashige and Skoog, 1962) supplemented with 0.05 mg L^-1^ 1-napthaleneacetic acid(NAA), 0.05 mg L^-1^ 2,4-dichorophenoxyacetic acid(2,4-D), 0.05 mg L^-1^ 6-benzylaminopurine(6-BA), 30g L^-1^ sucrose, 1g L^-1^ inositol and 0.8% (w/v) agar. The cultures were then placed in a light incubator at a temperature of 26°C with a 16 hour light cycle for 2 weeks.

### 2.3 Plant cell subculture

Induced cells from each callus were inoculated into solid MS medium (Murashige and Skoog, 1962) supplemented with NAA(0.05 mg L^-1^), 2,4-D(0.05 mg L^-1^), 6-BA(0.05 mg L^-1^), 30g L^-1^ sucrose, 1g L^-1^ inositol and 0.8% (w/v) agar and subsequently placed in a light culture chamber maintained at a temperature of 26°C under a 16 hour light cycle for two weeks (with subcultures every two weeks).

### 2.4 Plant cell suspension culture

*G. uralensis* cells cultured continuously for 20 generations with good growth conditions were selected as experimental materials. The cells were inoculated with a seeding amount of 10% (5 g of solid cells per 50 mL of culture medium) in 100 mL of MS liquid medium (Murashige and Skoog, 1962) supplemented with NAA(0.05 mg L^-1^), 2,4-D(0.05 mg L^-1^), 6-BA(0.05 mg L^-1^), 30g L ^-1^sucrose and 1 g L^-1^ inositol, at 25°C and 120 rpm for 5 days.

### 2.5 Determination of browning standard curve

PPO partial purification was performed using the method of Dong et al. (2016), and then catechol solutions with gradients of 0, 20, 40, 80, and 100 μg/mL were prepared. Purified PPO (100 ml) was added to catechol solution (3 ml) and incubated in a water bath at 25°C for 72 h. The absorbance values were determined at 410 nm and standard curves were plotted.

### 2.6 Measurement of cell browning degree

**Figure 1:**
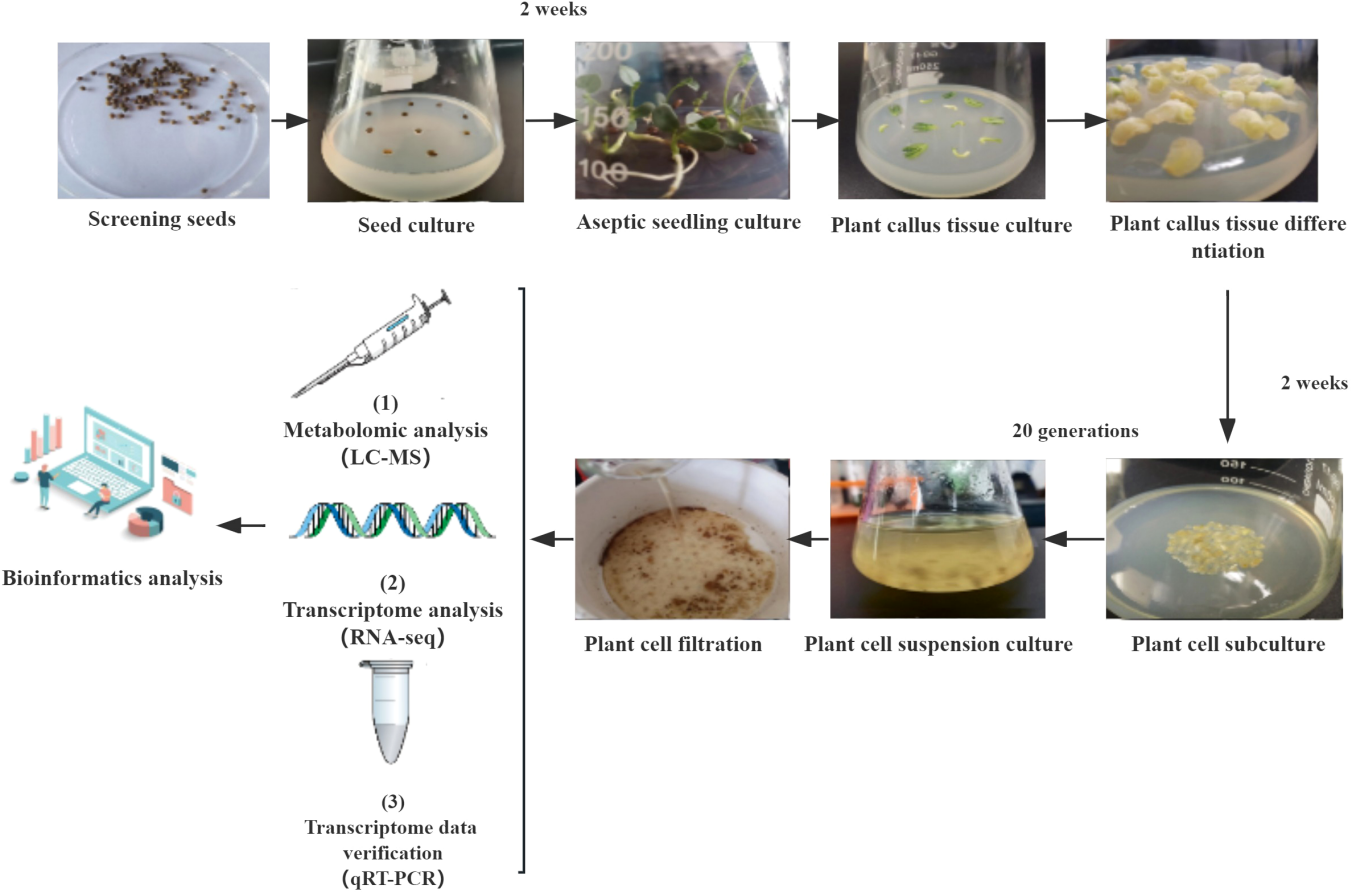
Metabolome and transcriptome workflow analysis.

**Figure 2:**
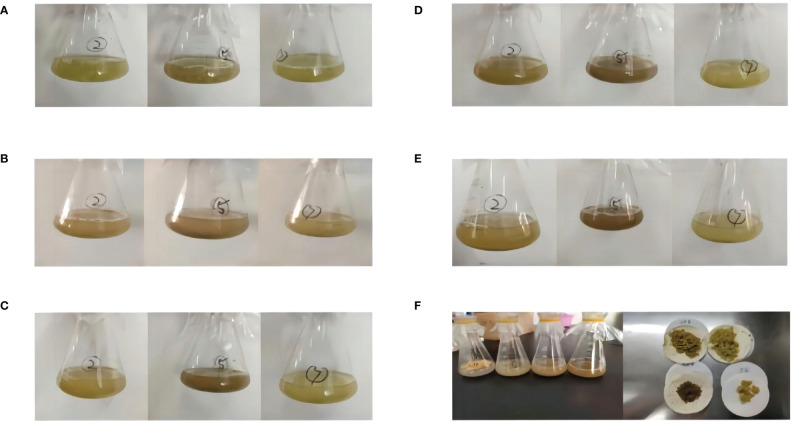
Changes in *G. uralensis* cell suspension culture for 5 days. **(A)** first day of suspension culture. **(B)** second day of suspension culture. **(C)** third day of suspension culture. **(D)**fourth day of suspension culture. **(E)** fifth day of suspension culture. **(F)** Collected cell samples.

The absorbance value of the cell culture solution after filtration was determined at 410 nm and calculated based on the standard curve, enabling the classification of the filtered cells into normal (5.04-5.06) and browned (5.08-5.10) categories for subsequent metabolome and transcriptome analysis (**Figures 1**, **2**) (Dong et al., 2016).

### 2.7 Extraction and analysis of metabolites in UHPLC-MS/MS

Metabolome analyses were commissioned to Qingdao Science Innovation Quality Testing Co., Ltd. (Qingdao, China), and normal *G. uralensis* cells were used as the control, while three biological replicates were set up.

The sample preparation procedure was conducted as follows: 100 mg of uniformly mixed sample was placed into a 2 ml centrifuge tube, and add 1 ml 70% methanol and 3 mm steel beads, and shaken and crushed for 3 minutes on a tissue lyser (JXFSTPRP-48,70Hz). After sample cooling, Ultrasonication at 40 Hz for 10 min, centrifugation at 4°C, 12000 rpm for 10 min. The resulting supernatant was diluted 2-100 times, passed through a 0.22 μm PTFE filter, and then injected into the LC-MS/MS system for analysis.

UHPLC-MS/MS analysis was performed using the Vanquish UHPLC system (Thermo Fisher Scientific GmbH, Germany) coupled with the Q Exactive™ HF mass spectrometer (Thermo Fisher Scientific GmbH, Germany) from Qingdao Science Innovation Quality Testing Co., Ltd (Qingdao, China). Chromatographic separation was conducted using a Zorbax Eclipse C18 column (100 mm×2.1 mm, 1.8 μm), Column temperature 30°C, Flow rate 0.3 mL/min; Mobile phase A was 0.1% formic acid in water (0.1% FA in Water), while mobile phase B was 100% acetonitrile. The sample volume was set at 2 μL, and the automatic sampler temperature was maintained at 4°C. The gradient elution program for the mobile phase is as follows: 0-2 min, 5% B, 95% A; 2-6 min, 30% B, 70% A; 6-7 min, 30% B, 70% A; 7-12 min, 78% B, 22% A; 12-14 min, 78% B, 22%A; 14-17 min, 95% B, 5% A; 17-20 min, 95% B, 5% A; 20-21 min, 5% B, 95% A; 21-25 min, 5% B, 95% A. The Q Exactive™ HF mass spectrometer in positive/negative mode has a heater temperature of 325°C, a sheath gas flow rate of 5 arb, an auxiliary gas flow rate of 15 arb, a purge gas flow rate of 1 arb, an electrospray voltage of 3.5 KV, a capillary temperature of 330°C, and an S-Lens RF Level of 55%. Substance analysis and identification are performed using a first-level full scan (m/z range: 100~1500) and data-dependent secondary mass spectrometry (dd-MS2; TopN = 5).

### 2.8 Data processing and metabolite identification

Compound Discoverer 3.3 was used to perform retention time correction, peak identification, peak extraction, peak integration, and peak alignment, on raw data files generated by UHPLC-MS/MS. Simultaneously, Substance identification using various databases including the Thermo mzCloud online database, the Thermo mzVault local database, and the ChemSpider database. OPLS-DA model calculations were conducted using SIMCA software (V14.1) to filter out orthogonal variables in metabolites unrelated to categorical variables and separately analyze non-orthogonal and orthogonal variables to obtain more reliable information regarding intergroup differences in metabolites and their association with browning phenomenon. Differential metabolite screening was performed using a threshold of |log2(Fold Change)|≥1, P-value ≤ 0.05, and VIP>1. Volcano plots were used to represent the differences and significance in expression levels of metabolites between the control group and the browning group. Cluster analysis heat maps were used to observe differential characteristics of metabolite data. KEGG pathway enrichment analysis was carried out using KEGG database (www.kegg.jp/kegg/pathway.html)and MetaboAnalystR to annotate differential metabolites and explore related metabolic pathways involved in the browning process. Furthermore, the volcano plot allows for rapid visualization of the differences and significance of metabolite expression levels between the two groups. Cluster analysis heat maps were used to identify characteristic features of differential metabolite data, while differential metabolite annotation and KEGG pathway enrichment were performed using MetaboAnalystR and the KEGG database (www.kegg.jp/kegg/pathway.html) to explore the specific differentially expressed metabolites and their associated metabolic pathways.

### 2.9 Transcriptome sample preparation, library construction and sequencing

The test samples comprised normal and browning cells, which underwent filtration. Three biological replicates were conducted for each set of experiments. Transcriptome analysis was outsourced to Shanghai Majorbio Bio-Pharm Technology Co., Ltd. Total RNA was extracted and isolated from the samples using the SDS method, while the concentration and purity of total RNA were assessed using Nanodrop2000(LabTech, USA), the RNA integrity was confirmed by agarose gel electrophoresis, and RIN values were determined using Agilent 2100(Agilent, USA). Qualified RNA served as input material for sample preparation following specific steps: firstly, magnetic beads with Oligo(dT) were employed to bind polyA through A-T base pairing to separate and enrich mRNA from total RNA, and then fragmentation buffer was added to randomly fragment the enriched mRNA into small fragments of approximately 300 bp in length via magnetic bead selection. Secondly, fragmented mRNA was subjected to reverse transcription using reverse transcriptase and random hexamers as primers to synthesize cDNA, followed by double-strand synthesis to form a stable duplex structure. An end repair mix was used to convert the sticky ends of the double-stranded cDNA into blunt ends and then an “A” base is added at the 3’ end to facilitate Y-junction formation. Library enrichment was achieved through PCR amplification and target bands were recovered from the PCR products on a 2% agarose gel, quantified by TBS380 (Picogreen), and clusters were generated by bridge PCR amplification using a cBot. Finally, the prepared library undergoes sequencing on the Illumina platform to obtain 150 bp paired-end reads.

### 2.10 Transcriptome data analysis

Statistical methods such as fastx-toolkit(V0.0.14) were used for statistical analysis and quality control on sequencing data, which could directly reflect the quality of sample library construction and sequencing from a macroscopic perspective. Reference genome indexes were constructed using Hisat2(v2.1.0) and mapped data were obtained by sequence alignment of paired-end clean reads with the reference genome (http://ngs-data-archive.psc.riken.jp/Gur-genome/index.pl). The mapped reads were assembled and spliced with StringTie (V2.1.2) and compared with known transcripts to obtain transcripts without annotation information, and then potential new transcripts were functionally annotated. Considering transcript count, transcript length, and expressed gene count in the sample, FPKM was calculated employing Rsem(V1.3.3). Samples were analyzed for differential expression of genes/transcripts between samples using DESeq2 (v1.24.0). Cluster analysis R package (3.24.3) was used to detect enrichment of differential genes in the KEGG pathway.

### 2.11 qRT-PCR validation

Normal and browning cells were used as test materials for qRT-PCR validation.

Firstly, total RNA was extracted and used as the sample for cDNA reverse transcription following the manufacturer instructions using a reverse transcription kit(TransGen Biotech, Beijing, Biotechnology, China). Two pairs of specific primers were designed for each gene using Primer 5.0(**Supplementary Table 1**). The obtained cDNA from reverse transcription served as a template for PCR amplification to screen for primers. The PCR reaction system consisted of 0.8 μL primer, 10 μL 2×Taq PCR Master Mix, and 1 μL cDNA in a final volume of 20 μL with double-distilled water. The PCR reaction procedure included pre-denaturation at 95°Cfor 4 min, denaturation at 95°C for 30 s, annealing for 30 s, and extension at 72°C for 1 min30 s; a total of thirty-four cycles, followed by an additional extension step at 72°C for 10 min. Finally, the PCR products were stored at 4°C, and optimal primers for qRT-PCR were determined through electrophoresis on a 1% agarose gel. The transcriptome sequencing results were then validated by qRT-PCR analysis using a qRT-PCR kit(Takara, Dalian, Biotechnology, China) and qRT-PCR instrument(Thermo Fisher Scientific, USA).

### 2.12 qRT-PCR result processing

Origin 2018 software(Microcal Software, Northampton, MA) was utilized for generating charts and graphics, while Photoshop software(Adobe, San Jose, California, United States) was used for image processing.

## 3 Results

### 3.1 Metabolome analysis of normal cells and browning cells

To investigate the metabolic accumulation changes in normal cells and browning cells following solid-liquid transformation samples were analyzed by UHPLC-MS/MS.

**Figure 3:**
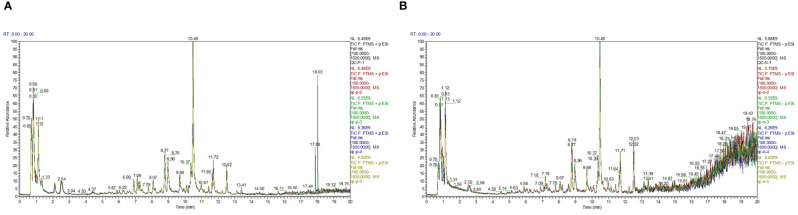
The total ion current (TIC) plots. **(A)** Overlapping TIC of the quality control sample under positive ion mode. **(B)** Overlapping TIC of the quality control sample under negative ion mode.

**Figure 4:**
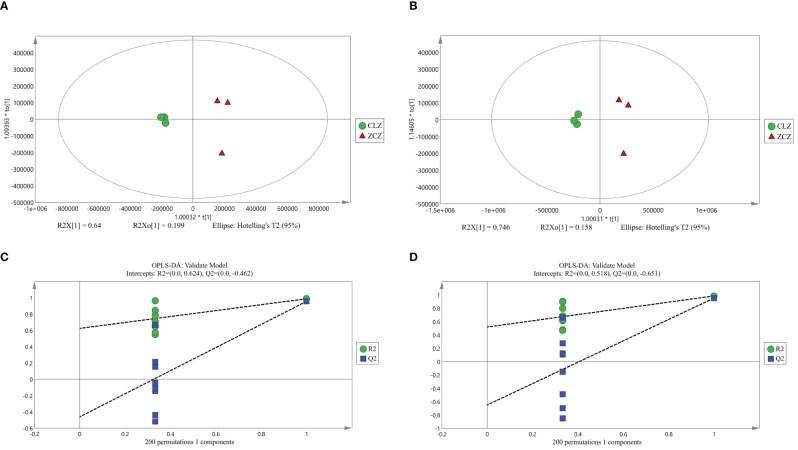
OPLS-DA score plot and validation plot. **(A)** OPLS-DA score plot under positive ion mode. **(B)** OPLS-DA score plot under negative ion mode. **(C)** OPLS-DA model validation plot under positive ion mode. **(D)** OPLS-DA model validation plot under negative ion mode. (CLZ stands for browning cell; ZCZ stands for normal cells).

Firstly, the reproducibility of the analytical process was examined by total ion current (TIC) plots overlap analysis. As depicted in **Figure 3**, the peak intensities and retention times of quality control samples (QC) were found to significantly overlap in both positive and negative ion modes, indicating excellent machine stability with minimal variations attributable to machine errors. Secondly, a total of 719 metabolites were detected across all samples, which were further subjected to orthogonal partial least squares-discriminant analysis OPLS-DA for variable assessment in both positive and negative ion modes. Among them, the model explanation ratios R2X were 0.839 and 0.904, while R2Y values were 0.987 and 0.984 respectively. Additionally, the model prediction abilities Q2 were determined to be 0.956 and 0.953 for positive and negative ion modes respectively, with all three indicators approaching unity (**Figure 4**). These results indicate that both models are stable, reliable, and excellent in performance. Furthermore, a permutation test using OPLS-DA was conducted on the X and Y variables (200 permutations). The intersection point of the Q2 regression line with the vertical axis was found to be less than zero(<0), indicating that overfitting did not occur in our model. Based on the aforementioned analysis, differential metabolites between normal and browning cells were selected based on criteria including |log2(Fold Change)|≥1, P-value ≤ 0.05, and VIP>1.

### 3.2 Differences in DAMs between normal and browning group cells

**Figure 5:**
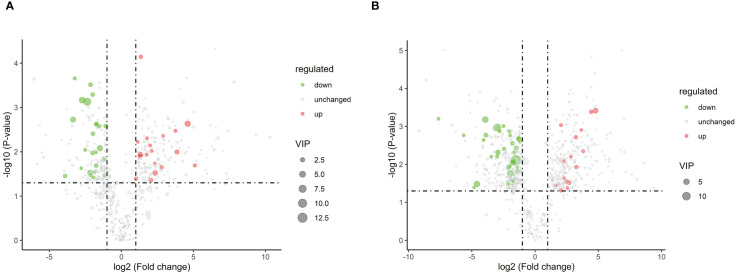
Volcano plots of DAMs. **(A)** represents the volcano plot of DAMs in positive ion mode. **(B)** represents the volcano plot of DAMs in negative ion mode. Each point in the figure represents a metabolite, where the size of the scatter plot represents the VIP value of the OPLS-DA model. Green points represent downregulated DAMs, red points represent upregulated DAMs, and black represents DAMs that were detected but with insignificant differences.

**Figure 6:**
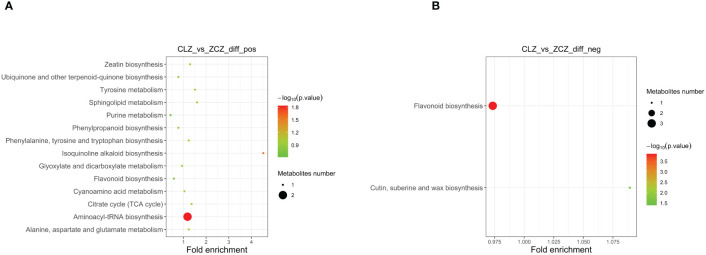
The enrichment degree of DAMs in the pathway is shown in this figure. **(A)** Enrichment degree of DAMs in positive ion mode. **(B)** Enrichment degree of DAMs in negative ion mode. The size of the points in the figure represents the number of significant DAMs enriched in the corresponding pathway.(CLZ stands for browning cells; ZCZ stands for normal cells).

To systematically identify DAMs in browning cells, three different criteria were used for screening. The first criterion involved the importance of VIP values derived from the first principal component in the OPLS-DA model, which indicates the strength of inter-group differences exhibited by corresponding metabolites and their impact on sample classification discrimination within the model. The second criterion was FC, representing the ratio of expression levels between groups. Lastly, P-values obtained through t-test hypothesis testing were used as our third criterion to indicate significant differences. Therefore, |log2 (Fold Change)| ≥ 1, VIP > 1, and P-value ≤ 0.05 were used as DAMs screening thresholds. Negative ion mode analysis identified a total of 49 DAMs, including 15 up-regulated and 34 down-regulated DAMs. Similarly, positive ion mode analysis revealed a total of 43 DAMs consisting of 19 up-regulated and 24 down-regulated (**Supplementary Tables 2, 3**) (**Figure 5**). Subsequently, the up-regulated DAMs were further classified into various categories including chalcone compounds, isoflavone compounds, imidazolepyrimidine compounds, purine nucleosides, organic oxides, carboxylic acids and their derivatives, benzene and its derivatives, flavonoids, 2-arylated benzofuran flavonoids, diazanaphthalenes, and fatty acyls. Notably, both positive ion mode and negative ion mode analysis revealed a significant increase in the content of flavonoids and isoflavonoids during cell browning. Annotation of DAMs in the normal and browning group was performed based on the KEGG pathway database (**Supplementary Tables 4, 5**). Additionally, KEGG metabolic pathway enrichment analysis was conducted to assess the distribution of DAMs within the metabolic pathways associated with browning cells. The results showed that under negative ion mode, four DAMs were assigned to two KEGG pathways in browning cells compared to normal cells. whereas under positive ion mode, eighteen DAMs were distributed across fourteen KEGG pathways. Furthermore, negative ion mode analysis showed significant enrichment of flavonoid biosynthesis as well as cutin, suberine, and wax biosynthesis in browning cells, while positive ion mode analysis indicated significant enrichment of aminoacyl-tRNA biosynthesis and isoquinoline alkaloid biosynthesis in browning cells (**Figure 6**).

### 3.3 Transcriptome analysis

#### 3.3.1 Transcriptome data quality assessment and counting of DEGs

**Figure 7:**
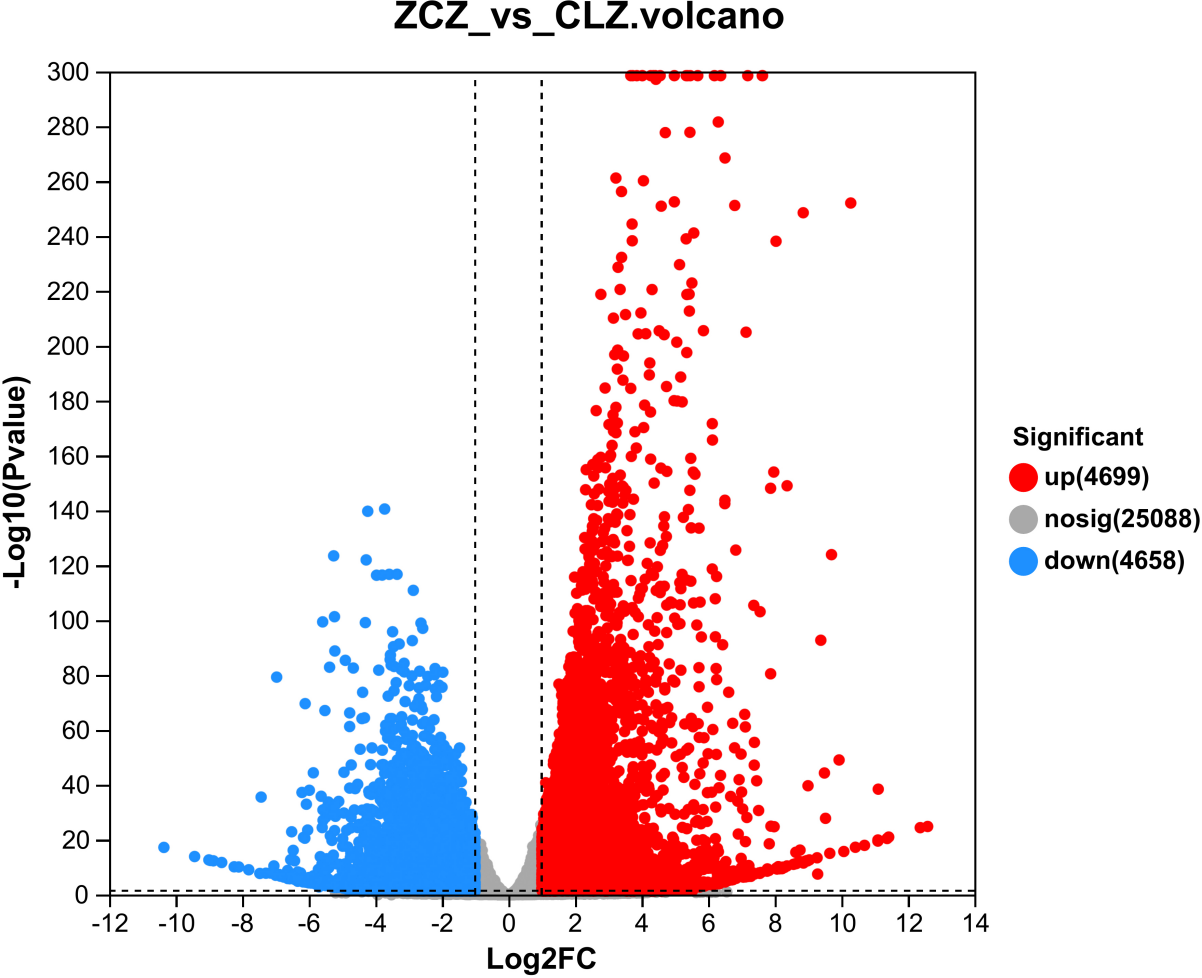
Volcano plot of DEGs. Red dots represent significantly up-regulated genes, green dots represent significantly down-regulated genes, and gray dots represent non-significantly differentially expressed genes.

Metabolome analysis revealed significant differences in the browning group of cells compared to the normal group. To identify the genes that regulate the browning of the cells, it was necessary to sequence the cells in the normal group and the browning group, and a total of 6G clean reads were obtained, with Q20>98%, Q30>94%, and the GC content between 46%~47%. Clean reads between 90% and 93% were mapped to the reference genome (http://ngs-data-archive.psc.riken.jp/Gur-genome/index.pl) using Hisat2 software(V2.1.0). **Supplementary Table 6** confirms that all samples met quality control standards. Based on this, transcriptome data were further analyzed. Our study identified a total of 4699 up-regulated genes and 4658 down-regulated genes in the browning group cells (**Supplementary Table 7**) (**Figure 7**).

#### 3.3.2 Effects of browning on the transcription factors of *G. uralensis* cells

**Figure 8:**
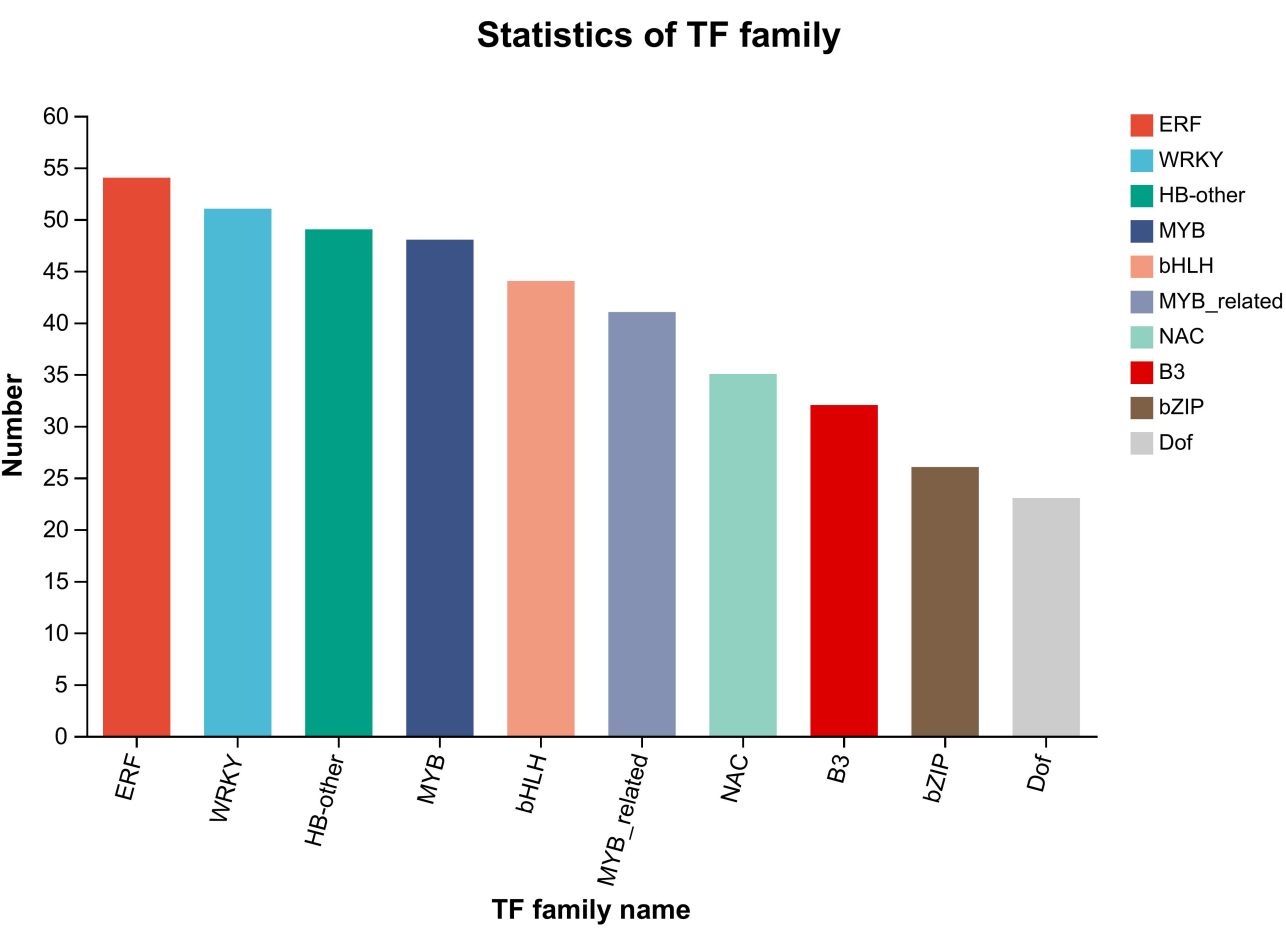
The number of transcription factor families and the genes they contain. The left-hand side denotes the genes. The right-hand side denotes the transcription factor families.

Transcription factors(TFs) play a crucial role in the adaptation of plants to changing environmental conditions (Li et al., 2019). Among the families of TFs identified in higher plants (Alvarez-Venegas et al., 2007), there are sixteen, including WRKY, bHLH, MYB, AP2/ERF, etc., which primarily regulate and control plant growth and metabolism (Ng et al., 2018). Within these TFs, the MYB transcription factor not only represents the largest proportion but also plays a pivotal role in flavonoid biosynthesis and phenylpropanoid pathway (Ma and Constabel, 2019). Therefore, it is essential to screen and identify TFs that govern secondary metabolite accumulation to understand the molecular mechanisms underlying metabolite production (Medina-Puche et al., 2014; Liu et al., 2017). We classified the screened DEGs into TFs families and identified a total of 43 TFs families in browning group cells(**Supplementary Table 8**). To explore the TFs families that mainly affect cell browning, the number of DEGs of the top 10 TFs families was compared in the normal and browning cell groups. In browning cells, the Top 10 TFs include ERF, WRKY, HB-other, MYB, bHLH, MYB-related, NAC, B3, bZIP, and Dof. The results showed that there are a substantial number of DEGs related to TFs in browning cells. For instance, EFR reached 54; WRKY reached 51; HB-other reached 49; MYB reached 48; bHLH reached44; MYB-related reached 41, NAC reached 35, B3 reached 32, bZIP reached 26, and Dof reached 23 (**Figure 8**). Subsequently, the results of metabolomics analysis were correlated with those of TFs, and it was found that the MYB transcription factor is a key factor regulating flavonoid synthesis during the browning of *G. uralensis*.

#### 3.3.3 Analysis of DEGs

**Figure 9:**
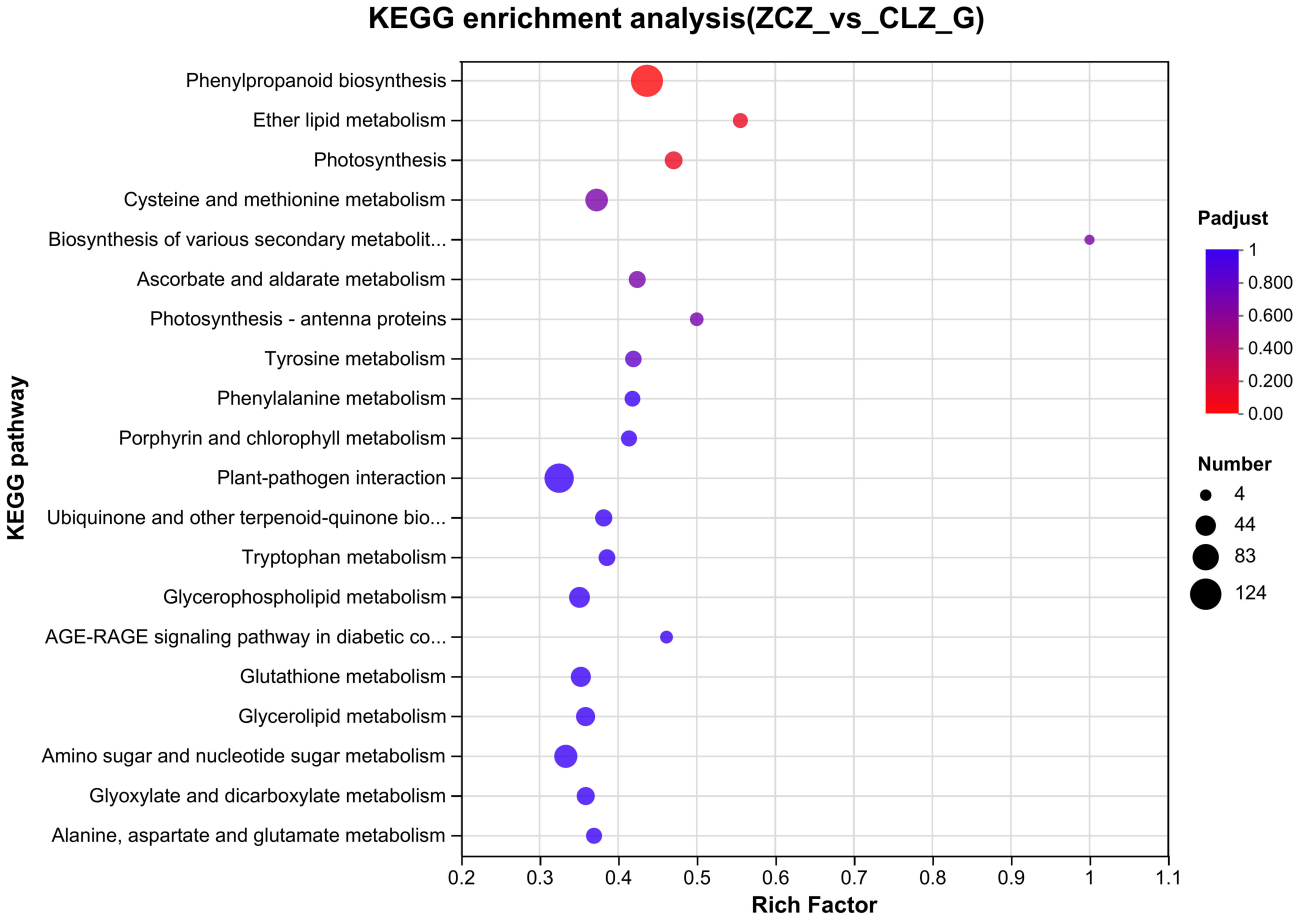
KEGG enrichment analysis of DEGs.

Based on the screening results of DEGs, KEGG pathway enrichment analyses were performed and identified the top 20 pathways based on a significance level of P<0.05 (**Figure 9**). Specific KEGG enrichment results are shown in **Supplementary Table 9**. Research has found that, compared with normal group cells, the DEGs of the browning group cells are significantly enriched in the phenylpropane biosynthesis pathway, photosynthesis, and ether lipid metabolism pathway. This indicates that phenylpropane biosynthesis pathway, photosynthesis, and ether lipid metabolism are all associated with the process of cell browning.

### 3.4 Integration analysis of metabolome and transcriptome

#### 3.4.1 KEGG enrichment analysis results of DEGs and DAMs are highly consistent

To determine the correlation of DEGs and DAMs on KEGG pathway between normal and browning group cells, metabolome and transcriptome KEGG enrichment analyses were performed. By comparing the significant KEGG pathways associated with DEGs and DAMs, we observed a high consistency between them.

#### 3.4.2 Integrative analysis of DEGs and DAMs affecting cell browning

The previous analysis of DAMs revealed a significant up-regulation of chalcones, isoflavonoids, and flavonoids in browning cells, all of which belong to phenolic compounds. Therefore, it can be inferred that these substances may serve as the primary substrates for cell browning. In higher plants, most of these substances are derived from the phenylpropane biosynthetic pathway. Further investigation showed that the synthesis of phenylalanine in this pathway relies on the upstream shikimate pathway. The starting materials for the shikimate pathway originate from phosphoenolpyruvate in the glycolytic pathway and erythrose 4-phosphate in the pentose phosphate pathway. To screen DEGs related to the synthesis of chalcone, isoflavone, and flavonoid synthesis the relevant KEGG pathways corresponding to these three DAMs were compared with those of the DEGs. 223 DEGs were compared with the key enzyme genes in the synthesis pathway of the above compounds(**Supplementary Table 10**). As a result, a total of 23 key enzyme genes were identified (**Supplementary Table 11**).

### 3.5 Validating the accuracy of transcriptome data through qRT-PCR

**Figure 10:**
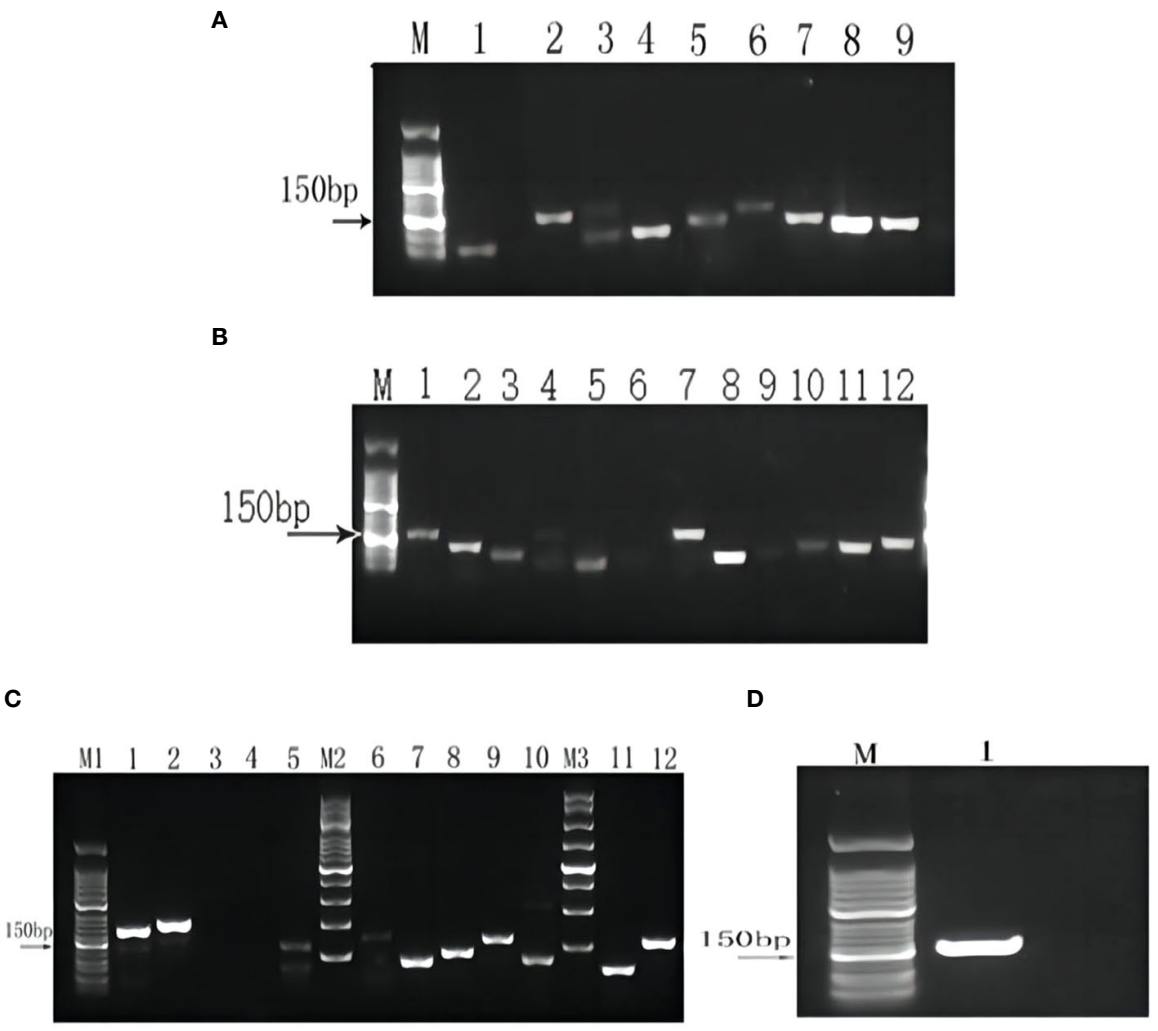
Primer screening. **(A)** M:50 bp DNA Ladder Marker; 1-9: *Gl1CHI1, Gl1CCR1, Gl1CCR2, Gl2CCR1, Gl1F3’H1, Gl1F3’H2, Gl2F3’H1, Gl2F3’H2, Gl3F3’H1*. **(B)** M:50 bp DNA Ladder Marker; 1-12: *Gl1PDT1, Gl1PDT2, Gl4CL1, Gl4CL2, GlIFR1, GlIFR2, GlCHS1, GlCHS2, Gl4F3’H1, Gl4F3’H2, GlC4H1, GlC4H2*. **(C)** M1: 50 bp marker; 1-5: *Gl3CHI1, Gl3CHI2, GlGPI1, GlGPI2, Gl2PDT1*; M2: 200 bp; 6-10: *Gl2PDT2, Gl24CL1, Gl24CL2, GlPK1, GlPK2*; M3: 250 bp DNA Ladder Marker; 11-12: *GlPAL1, GlPAL2*. **(D)** M1: 50 bp DNA Ladder marker; 1: *Gl3PDT1*.

**Figure 11:**
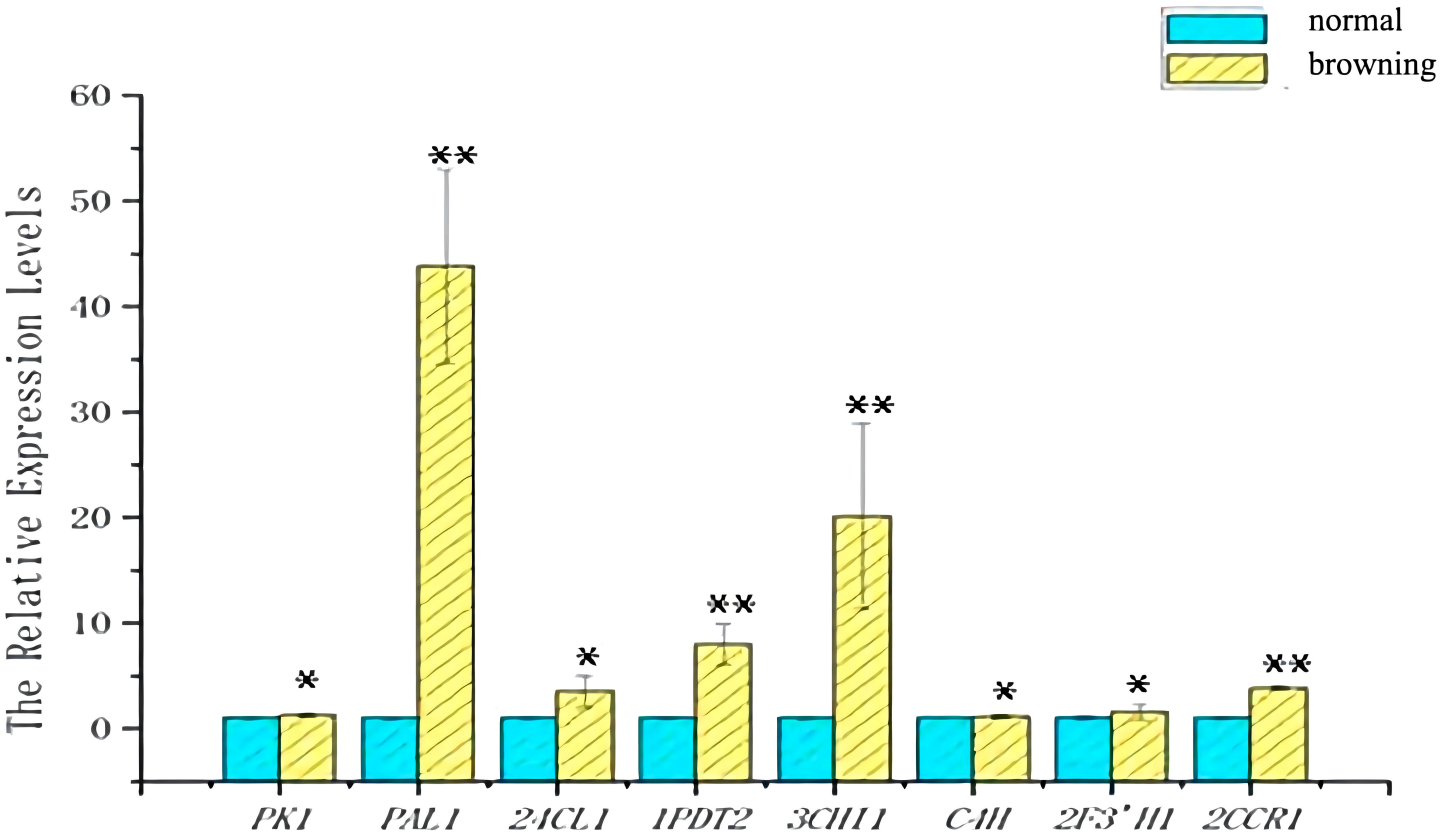
Expression of genes in normal and browning cells. Blue indicates the amount of gene expression in normal cells. Yellow indicates the amount of gene expression in browning cells. Statistical significance between treatment and control groups was tested using Duncan’s test (*p < 0.05; **p < 0.01), biological replicates n=3.

To verify the reliability of the transcriptome data, primer screening resulted in 17 genes(*Gl1CHI*, *Gl1CCR, Gl2CCR, Gl1F3’H, Gl2F3’H, Gl3F3’H, Gl1PDT, Gl4CL, GlCHS, Gl4F3’H, GlC4H, Gl3CHI, Gl2PDT, Gl24CL, GlPK, GlPAL, Gl3PDT*) were used in subsequent qRT-PCR experiments (**Figure 10**). The validation results revealed that eight key enzyme genes, including *GlPK, GlPAL, Gl24CL, Gl1PDT, Gl3CHI, GlC4H, Gl2F3’H, and Gl2CCR*, were expressed at higher levels in browning cells than in normal cells (**Figure 11**). It indicates that the transcriptome sequencing data are in good agreement with the qRT-PCR results, and these 8 key genes regulate cell browning.

## 4 Discussion

According to the World Health Organization (WHO), 80% of the world’s population relies on herbal medicine for primary health care, and nearly 25% of modern drugs originate from or are derived from natural plants. It has been found that secondary metabolites constitute the main medicinal constituents of medicinal plants, but these tissues contribute less than 1% to their medicinal activity (Suntar et al., 2017; Zafar et al., 2019; Elkordy et al., 2021). With the over-exploitation of medicinal plants, there is a growing challenge in the production of secondary metabolites. The plant cell suspension culture technique not only enables rapid production of identical or similar medicinal active compounds as those found in parent plants but also represents a simple and cost-effective biotechnology (Motolinía-Alcántara et al., 2021). Currently, this technology is widely used for cell culture of medicinal plants such as Taxus chinensis, Glycyrrhiza, Tripterygium wilfordii Hook, Pueraria, and Panax. It has been demonstrated to be the preferred technical approach for obtaining high-value medicinal ingredients like paclitaxel (Sharma and Zafar, 2016; Gauchan et al., 2021), resveratrol (Chastang et al., 2018; Jeong et al., 2020), and ginsenosides (Le et al., 2019). However, the occurrence of browning significantly hampers cell growth by impeding biomass accumulation and reducing secondary metabolite yield. This phenomenon poses a major bottleneck that severely restricts the industrial application of plant cell culture. Due to the inherent characteristics of plant cells and the complexity of suspension culture environments, there is currently limited understanding regarding the mechanism underlying browning in suspension systems. Furthermore, specific and effective control measures have not yet been proposed. Presently, traditional methods such as incorporating antioxidants, chelating agents, and adsorbents are primarily used to control browning. Although these approaches can partially control cellular browning to some extent, they fail to address the problem at its core. Moreover, their addition exerts toxic effects on cells themselves while also increasing economic costs.

The present study investigates the metabolome and transcriptome of normal and browning cells, elucidating the underlying material basis and molecular regulatory mechanisms of enzymatic browning in plant cells during enlarged culture. Metabolome analysis reveals significant variations in the levels of chalcones, flavonoids, and isoflavones in browning cells, with all up-regulated substances being enriched in four metabolic pathways: isoflavone biosynthesis; cutin, suberine and wax biosynthesis; aminoacyl-tRNA biosynthesis; and isoquinoline alkaloid biosynthesis. Transcriptome studies identify ERF, WRKY, HB-other, MYB, bHLH, MYB-related, NAC, B3, BZIP, and Dof as the top 10 transcription factors involved in cell browning. Of note is the key role of the MYB transcription factor in regulating flavonoid synthesis during *G. uralensis* cell browning. Additionally, a total of 223 differentially expressed genes (DEGs) were significantly enriched in the phenylpropane pathway, shikimic acid pathway, glycolysis pathway, and pentose phosphate pathway. Subsequently, these 223 DEGs were compared with key enzyme genes involved in chalcone, flavonoid, and isoflavone synthesis resulting in the identification of eight key genes (*Gl1CHI, Gl1CCR, Gl2CCR, Gl1F3’H, Gl2F3’H, Gl3F3’H, Gl1PDT, Gl4CL, GlCHS, Gl4F3’H, GlC4H, Gl3CHI, Gl2PDT, Gl24CL, GlPK, GlPAL, Gl3PD*) exhibiting higher expression levels in browning cells compared to normal cells. The transcriptome data was further validated through qRT-PCR analysis which confirmed agreement between the qPR-PCR results and transcriptome findings.

## Data availability statement

The original contributions presented in the study are included in the article/**Supplementary Files**, further inquiries can be directed to the corresponding authors.

## Author contributions

XYZ: Writing – original draft, Writing – review & editing. XL: Writing – original draft, Writing – review & editing. AB: Investigation, Writing – review & editing. XLZ: Formal analysis, Writing – review & editing. YL: Writing – original draft, Writing – review & editing. YX: Conceptualization, Writing – review & editing.
